# Examining the Gap between Science and Public Opinion about Genetically Modified Food and Global Warming

**DOI:** 10.1371/journal.pone.0166140

**Published:** 2016-11-09

**Authors:** Brandon R. McFadden

**Affiliations:** Department of Food and Resource Economics, University of Florida, Gainesville, Florida, United States of America; Utrecht University, NETHERLANDS

## Abstract

There is great uncertainty due to challenges of escalating population growth and climate change. Public perception that diverges from the scientific community may decrease the effectiveness of scientific inquiry and innovation as tools to solve these challenges. The objective of this study was to identify the factors associated with the divergence of public opinion from scientific consensus regarding the safety of genetically modified (GM) foods and human involvement in global warming (GW). Results indicate that the effects of knowledge on public opinion are complex and non-uniform across types of knowledge (i.e., perceived and actual) or issues. Political affiliation affects agreement with science; Democrats were more likely to agree that GM food is safe and human actions cause GW. Respondents who had relatively higher cognitive function or held illusionary correlations about GM food or GW were more likely to have an opinion that differed from the scientific community.

## Introduction

Science, which plays an important role in innovation [1] and biotechnology [2], does not operate in a vacuum. Particularly, it is unclear whether some of the general public is in agreement with much of the scientific community regarding the safety of genetically modified (GM) food and human involvement in global warming (GW). Gaps between science and public opinion regarding these issues can be burdensome because the ultimate decisions are not made solely by scientists or elected officials. Individuals have the ability to affect policy in the voting booth and the ability to affects markets by choosing certain foods or relatively climate-friendly goods and services. Thus, public opinion could theoretically reduce investments in public science in these areas.

Recently, the Pew Research Center surveyed scientists belonging to the American Association for the Advancement of Science (AAAS) and the U.S. general public to examine opinions about contemporary issues in *biomedical sciences*, and *climate*, *energy*, *space sciences* [3]. The majority of AAAS scientists agreed that it is safe to eat GM foods (88%) and that human activities cause GW (87%). While it appears that the scientists surveyed have reached a near consensus, the general public is not as convinced. The same study revealed that only 37% and 50% of U.S. adults believed that GM foods are safe to eat and GW is related to human activity, respectively. The 51 percentage point gap between AAAS scientists and public opinion about GM foods was the largest gap for *biomedical sciences* and the 37 percentage point gap for anthropogenic GW was the largest for *climate*, *energy*, *space sciences*. Obviously, these are two issues for which there is great dissonance between science and public opinion.

Because there is often a gap between science and public opinion [4,5,6], there is a need to better understand what factors affect the dissonance. The objective of this study was to determine the factors associated with public opinion that oppose scientific consensus regarding GM foods and human involvement in GW. The analysis is structured as follows: a background on factors associated with dissonance between science and public opinion is discussed, then a detailed methodology of the study is presented, followed by results and conclusions.

## Background

The gap between science and public opinion may be explained by a deficiency in knowledge by the general public [7]. Intuitively, it would seem likely that greater knowledge would be associated with being more agreeable with science. Indeed, individuals with greater actual knowledge are more agreeable science in general, however, individuals with greater actual knowledge become less agreeable when the issues are contentious [8]. The issues of GM food safety and anthropogenic GW are contentious. When examining the effects of knowledge on public opinion, knowledge is typically measured by asking participants a question about perceived knowledge [9,10,11,12], or actual knowledge is measured with assessment questions [8,13], or both perceived and actual knowledge are measured [14]. Previous research has concluded that concerns about the safety of GM food are affected by perceived knowledge affects [9] and actual knowledge [10]. Findings for how concerns about human involvement in GW are affected by perceived knowledge are not consistent [11,12] but the effect of actual knowledge has been found to be powerful [13].

Public opinion about issues, especially those that can be affected by a matter of vote, can become politically polarized. Political affiliation is one of the most consistent predictors of concern about GW [15], such that more Democrats accept human involvement in GW than do Republicans [16]. Republicans are sometimes characterized as science deniers [17,18] or as incapable of fully understanding the possible impacts of GW [19]. The political division is less defined on the safety of GM foods. Other types of complexities associated with GM food safety and GW include corporate control in the production of food and government intervention, respectively. The Anti-Reflexivity Thesis attempts to account for these complexities by postulating that Republicans (Democrats) are more (less) likely to agree with science that provides innovations for economic production and less (more) likely to agree with science that identifies negative impacts of economic production [20]. According to this thesis, Republicans should be relatively more agreeable towards GM foods and Democrats should be relatively more agreeable to anthropogenic GW.

Cognitive characteristics may cause some of the general public to form beliefs that disagree with scientists. Computational constraints often require consumers to employ heuristics, or rules of thumb, which can lead to biases [21]. Exploration into the psychology of beliefs and deviation from normative decision-making prompted the partition of cognitive function. Stanovich and West [22] formally defined the two modes of cognitive function as System 1 and System 2, and the systems can be thought of more generally as intuition and reasoning, respectively [23]. Individuals who rely on System 1 make more emotionally charged decisions, while individuals who rely on System 2 make more analytical decisions. Tendencies to make emotionally charged decisions may have implications for beliefs formed despite evidence to the contrary.

There are differences in the demographic characteristics associated with the gap between science and public opinion concerning GM food safety and GW. Younger, more educated, and male are associated with being more accepting of GM foods [14]. Younger and female are associated with being more concerned about GW [11,16], but education is not [24]. Income does not appear to be related to opinions about either issue.

A phenomenon that is sometimes observed when there is discourse between public and scientific opinion is illusory correlation. This occurs when an individual believes a correlation exists between two events that are uncorrelated, or are correlated but to a lesser extent than believed, or are correlated in the opposite direction than believed [25]. Illusory correlations may contribute to the formation of false hypotheses [21]. One example of illusory correlation is the causal connection between vaccinations and autism. Despite findings that there is no increased risk for autistic disorder due to exposure to vaccines [26], some of the general public continue to believe that vaccinations will cause autism. Similarly is the concern about the association between GM food and autism. It is impossible to know whether illusory correlations cause the formation of false hypotheses, or illusory correlations are formed to protect false hypotheses.

## Methods

### Respondents and Survey Overview

This study was approved by the institutional review board at Oklahoma State University. The survey was conducted online and participants consented before taking the survey. Consent was obtained and recorded using Qualtrics (survey design and data collection software).

To address the research questions, an internet survey was developed and administered to a representative sample of the U.S. population. The survey was sent to a sample of 961 respondents enrolled in an online panel maintained by Qualtrics and their associated partners. Five respondents did not respond to all questions and therefore were excluded from this study. The survey was fielded from April 24, 2013 through April 27, 2013. Qualtrics prescreened respondents by sex, education, and income to ensure the sample was representative of the U.S population. According to the 2012 U.S. Census Bureau, females represented 50.8% of the population, 28.2% of persons age 25+ held a Bachelor’s degree, and the median household income was $52,762. The survey sample closely matched the 2012 population statistics. Fifty-one percent of the survey sample was comprised of females (*SD* = 0.50), 29% percent held a Bachelor’s degree (*SD* = 0.46), and the median income category was $40,000 to $59,999. However, the median age of the sample was 26 years of age, which is younger than the U.S. median of 37.2.

After consenting to take the survey, respondents were asked questions about GM food and GW that were arranged in separate blocks; the blocks were counterbalanced across respondents to eliminate an order effect. Within each block, questions were asked in the following order: 1) questions that measured a respondent’s beliefs about the safety of GM foods or human involvement in GW; 2) a question that determined whether the respondents believed scientific research supported a belief; 3) questions that determined whether a respondent held illusory correlations about GM food or GW; and 4) questions that determined objective knowledge of GM foods or GW.

Respondents completed the Cognitive Reflection Test (CRT) introduced by Frederick [27] after answering the two blocks of questions about GM food and GW. The CRT is a three-question test designed to generate incorrect intuitive answers and has been used to measure the ability of an individual to engage in higher forms of reasoning. Recent studies suggest that the CRT is superior to self-reported measures and can predict performance on rational-thinking tasks [28] and susceptibility to cognitive biases [29]. Respondents finished the survey by answering questions that determined political party affiliation and demographic characteristics. The specific questions asked are provided in the supplementary material for the interested reader.

### Variables and Summary Statistics

A respondent’s belief about an issue was measured by asking the level of agreement with a statement about the safety of GM food and GW. The statements presented were: “Genetically modified crops are safe to eat” and “The Earth is getting warmer because of human actions,” respectively. Respondents chose a level of agreement for each statement from a symmetric five-point scale with the following response options: Strongly Disagree, Disagree, Neither Agree nor Disagree, Agree, and Strongly Agree.

**Fig 1:**
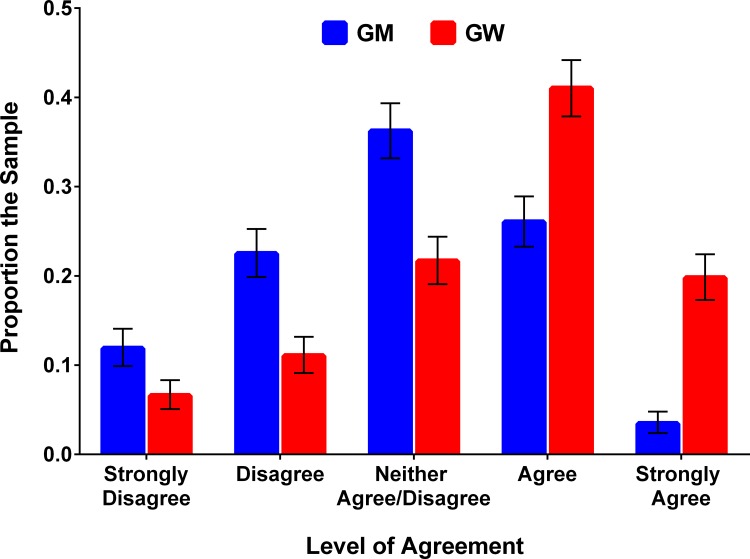
Relative Frequencies of Beliefs about the Safety of Genetically Modified Foods (GM) and Human Involvement in Global Warming (GW).

Fig 1 shows the relative frequencies of respondent beliefs about the safety of GM foods or human involvement in GW. Approximately 12%, 23%, 36%, 26%, and 3% of respondents strongly disagreed, disagreed, neither agree nor disagree, agreed, and strongly agreed that GM foods were safe to eat, respectively. Conversely, approximately 7%, 11%, 21%, 41%, and 20% of respondents strongly disagreed, disagreed, neither agree nor disagree, agreed, and strongly agreed that human activities cause global warming, respectively. As shown in Fig 1, respondents were more likely to agree that human actions cause GW than that GM food is safe to consume (*P* < 0.01, paired t-test).

These findings are reasonably similar to those found by Pew Research Center [3]. Compared to the Pew Research Center, the present study found lower support for safety of GM food (29% vs. 37%) and higher support for human involvement in GW (61% vs. 50%). The small differences in responses between the two surveys could be attributed to differences in the way the questions were asked or the response categories used. For example, to measure GW beliefs, Pew Research Center asked which of four statements about the earth’s temperature came closest to the respondent’s view. The four response categories were: 1) “the earth is getting warmer mostly because of human activity such as burning fossil fuels” (49% picked this option), 2) “The earth is getting warmer mostly because of natural patterns in the earth’s environment” (36% picked this option), 3) “There is no solid evidence that the earth is getting warmer” (11% picked this option), and 4) “Don’t know” (4% picked this option).

What is not obvious by examining the relative frequencies of respondent beliefs about the safety of GM foods or human involvement in GW, as shown in Fig 1, is joint frequency of disagreement with the scientific consensus about both issues. It is possible that some people are more likely to not agree with science in general, or at least for both of the issues presented within. To determine whether some respondents were in disagreement with science about both issues, beliefs for each issue were categorized into one of the following groups: *Disagree* or *Do not disagree*. Respondents who chose either Strongly Disagree or Disagree as the level of agreement for a given issue were placed in the *Disagree* category, while respondents who chose Neither Agree nor Disagree, Agree, or Strongly Agree were placed in the *Do not disagree* category. The joint frequencies of disagreement about the GM food safety and anthropogenic GW are shown in Table 1. The majority of respondents (54%) did not disagree with science about either issue, while a small percentage of respondents (7%) disagreed about both. Twenty-eight percent of respondents disagreed that GM foods were safe to eat but agreed that human actions are causing GW; 11% responded in the opposite manner.

**Table 1:** Joint Frequency of Disagreement with Scientific Consensus about the Safety of Genetically Modified Foods and Human Involvement in Global Warming.

	Human Involvement in Global Warming		
Safety of Genetically Modified Foods	*Do not disagree*	*Disagree*	
*Do not disagree*	522 (54%)	104 (11%)	626 (63%)
*Disagree*	265 (28%)	64 (7%)	329 (37%)
	787 (82%)	168 (18%)	955 (100%)

Note: Percentages in parentheses are relative frequencies.

Explanatory variables were created using responses from the previous questions. Descriptions and means of explanatory variables are shown in Table 2. On average, respondents had significantly higher levels of perceived knowledge about GW than GM food (*P* < 0.01, paired t-test), but significantly lower levels of actual knowledge (*P* < 0.01, paired t-test). Furthermore, illusory correlation was significantly higher for GM food than GW (*P* < 0.01, paired t-test).

**Table 2:** Descriptions and Means of Explanatory Variables.

Explanatory Variables	Descriptions	Means
*Perceived knowledge GM*	An integer variable ranging from 1 (strongly disagree) to 5 (strongly agree), determined by the level of agreement that scientific research supported a belief about the safety of GM foods.	3.278
*Perceived knowledge GW*	An integer variable ranging from 1 (strongly disagree) to 5 (strongly agree), determined by the level of agreement that scientific research supported a belief about human involvement in GW.	3.662
*Actual knowledge GM*	An integer variable ranging from 0 to 3, determined by the number of correctly answered true/false questions about GM foods.	2.050
*Actual knowledge GW*	An integer variable ranging from 0 to 3, determined by the number of correctly answered true/false questions about GW.	1.062
*Strong Democrat*	1 if a respondent self-identified as a Strong Democrat, 0 otherwise.	0.094
*Democrat*	1 if a respondent self-identified as a Democrat, 0 otherwise.	0.192
*Lean Democrat*	1 if a respondent self-identified as an Independent Lean Democrat, 0 otherwise.	0.106
*Independent*	1 if a respondent self-identified as an Independent, 0 otherwise.	0.221
*Lean Republican*	1 if a respondent self-identified as an Independent Lean Republican, 0 otherwise.	0.080
*Republican*	1 if a respondent self-identified as a Republican, 0 otherwise.	0.149
*Strong Republican*	1 if a respondent self-identified as a Strong Republican, 0 otherwise.	0.063
*CRT*	An integer variable ranging from 0 to 3, determined by the number of correctly answered Cognitive Reflection Test questions.	0.319
*Age*	Age in years.	26.752
*Bachelors*	1 if Bachelor’s degree or higher, 0 otherwise.	0.293
*Female*	1 if female, 0 if male.	0.512
*Income*	An integer variable ranging from 1 to 8, used to represent income categories (1 = $0–19,999, 2 = $20,000-$39,999…8 = $140,000 or more).	3.355
*Illusory correlation GM*	An integer variable ranging from 3 (strongly disagree) to 15 (strongly agree), determined by the sum of three level of agreement questions measuring illusory correlations about GM foods.	8.981
*Illusory correlation GW*	An integer variable ranging from 3 (strongly disagree) to 15 (strongly agree), determined by the sum of three level of agreement questions measuring illusory correlations about human involvement in GW.	7.715

### Econometric Models

Individual differences that affected the level of agreement about the safety of GM foods and human involvement in GW were examined by estimating an ordered probit model for each issue. An ordered probit is the appropriate model for regressing the level of agreement on individual characteristics because level of agreement is a limited, ordered choice possibility that is measured discretely across individuals. The mathematical description of the ordered probit provided below closely follows Greene [30].

Respondents level of agreement, denoted as y* and illustrated in Fig 1, depends on the explanatory variables measured, denoted as ***x***, and unobservable factors, denoted as *ε*, and can be expressed by:

$$y^{*}=x^{\prime }\beta +\varepsilon ,$$ (1)

where ***β*** are unknown parameters to be estimated. While we do not observe y*; however, we do observe *y* which is the level of agreement that most closely represents a respondent’s belief about the safety of GM foods and human involvement in GW. Therefore,

$$y=\left\{ \begin{array}{ll} 0\Rightarrow Stongly\;Disagree & if\;y^{*}\leq 0\\ 1\Rightarrow Disagree & if\;0<y^{*}\leq \mu_{1}\\ 2\Rightarrow Neither\;Agree/Disagree & if\;\mu_{1}<y^{*}\leq \mu_{2}\\ 3\Rightarrow Agree & if\;\mu_{2}<y^{*}\leq \mu_{3}\\ 4\Rightarrow Stongly\;Agree & if\;\mu_{3}<y^{*} \end{array}\right.,$$ (2)

where the *μ*’s are additional parameters to be estimated. Assuming that *ε* is independent and identically distributed normally across observations, then the probabilities that *y* = 0,…4 are

$$\begin{array}{l} Prob(y=0|x)=\Phi (-x^{\prime }\beta )\\ Prob(y=1|x)=\Phi (\mu_{1}-x^{\prime }\beta )-\Phi (-x^{\prime }\beta )\\ Prob(y=2|x)=\Phi (\mu_{2}-x^{\prime }\beta )-\Phi (\mu_{1}-x^{\prime }\beta )\\ Prob(y=3|x)=\Phi (\mu_{3}-x^{\prime }\beta )-\Phi (\mu_{2}-x^{\prime }\beta )\\ Prob(y=4|x)=1-\Phi (\mu_{3}-x^{\prime }\beta ) \end{array},$$ (3)

where Φ denotes the cumulative normal distribution.

The relationship between illusory correlations and level of agreement about GM food safety and anthropogenic GW were also of interest. However, the illusory correlation variables were excluded as independent variables in the estimation of the GM and GW ordered probit models because of possible simultaneity. Therefore, correlations coefficients were estimated to test the null hypothesis that there was not a relationship between the levels of agreement and illusory correlations.

To further examine the heterogeneity in disagreement with scientific consensus, the joint distribution of the *Disagree* and *Do not disagree* categories for the GM and GW models, as shown in Table 1, was used as dependent variables in the estimation of a multinomial logit. More specifically, binary coding was used to create the following: *Do not disagree GM & GW*, *Disagree GM & do not disagree GW*, *Do not disagree GM & disagree GW*, and *Disagree GM & GW*. A multinomial logit is the appropriate model because the dependent variable is a discrete measure for an individual across limited, unordered choice possibilities. The mathematical model of the disagreement with scientific consensus closely follows Greene [30], and is

$$\begin{array}{c} Prob(Y_{i}=j|w_{i})=\frac{exp(w_{i}^{\prime }\alpha_{j})}{\sum_{j=0}^{3}exp(w_{i}^{\prime }\alpha_{j})},j=\left\{ \begin{array}{l} 0\Rightarrow Do\;not\;disagree\;GM\;\&\;GW\\ 1\Rightarrow Disagree\;GM\;\&\;do\;not\;disagree\;GW\\ 2\Rightarrow Do\;not\;disagree\;GM\;\&\;disagree\;GW\\ 3\Rightarrow Disagree\;GM\;\&\;GW \end{array}\right. \end{array},$$ (4)

where ***w***_*i*_ denotes a vector of explanatory variables and ***α***_*j*_ are parameters to be estimated. The same explanatory variables used in the ordered probit models were used in the multinomial logit model. However, due to identification problems, the Democrat and Republican indicator variables were collapsed to create the variables *Combined Democrat* and *Combined Republican*.

## Results

Marginal effects, rather than coefficients, are reported for the ordered probit models and multinomial logit model. A marginal effect for an independent variable indicates how the probability of being in an agreement category–for the ordered probit models–or the probability of being in a joint distribution category–for the multinomial logit–changes for a given change in an explanatory variable.

The marginal effects for the GM foods ordered probit model are displayed in Table 3. Increases in either *Perceived knowledge* or *Actual knowledge* decreased the probability of being in the Strongly Disagree and Disagree categories and increased the probability of being in the Agree and Strongly Agree categories. Thus, both subjective and objective knowledge affected the level of agreement that GM food is safe to consume, and higher levels of knowledge were associated with being in agreement with scientific consensus. The marginal effects for the political affiliation variables indicated that respondents who identified as more extreme Democrats or Republican agree somewhat more about the safety of GM foods than those who do not identified as less extreme. *Strong Democrat* and *Democrat* were less likely to be in the Strongly Disagree and Disagree categories and more likely to be in the Agree and Strongly Agree categories. *Strong Democrat* were also less likely to be in the Neither Agree/Disagree category. *Strong Republican* and *Republican* were less likely to be in the Strongly Disagree, and *Strong Republican* was also less likely to be in the Disagree category and more likely to be in the Agree and Strongly Disagree categories. The only other independent variable that had significant marginal effects in the GM ordered probit model was *Female*. A respondent who was female was more likely to be in the Strongly Disagree and Disagree categories and less likely to be in the Agree and Strongly Agree categories.

**Table 3:** Marginal Effects from the Genetically Modified Foods Ordered Probit Model.

	Dependent Variable: Level of Agreement				
Explanatory Variables	Strongly Disagree	Disagree	Neither Agree/Disagree	Agree	Strongly Agree
*Perceived knowledge*	-0.709***	-0.086***	0.010	0.129***	0.017***
*Actual knowledge*	-0.041**	-0.050***	0.006	0.075***	0.010***
*Strong Democrat*	-0.706**	-0.118***	-0.044*	0.190***	0.043**
*Democrat*	-0.041**	-0.057**	-0.003	0.087**	0.014*
*Lean Democrat*	-0.018	-0.024	0.001	0.037	0.005
*Independent*	-0.023	-0.030	0.001	0.045	0.007
*Lean Republican*	-0.004	-0.005	0.001	0.008	0.001
*Republican*	-0.032*	-0.044	-0.001	0.067	0.010
*Strong Republican*	-0.044**	-0.066**	-0.011	0.103*	0.018
*CRT*	0.001	0.001	-0.00	-0.002	0.000
*Age*	0.000	0.000	0.000	0.000	0.000
*Bachelors*	-0.020	-0.026	0.002	0.039	0.005
*Female*	0.060***	0.072***	-0.008	-0.108***	-0.015***
*Income*	0.000	0.000	0.000	0.000	0.000

Note: Estimates are from an ordered probit model using 955 observations and a log likelihood function of -1,231. Single, double, and triple asterisks (*, **, ***) indicate statistical significance at the 10%, 5%, and 1% level.

The marginal effects for the GW ordered probit are displayed in Table 4. Subjective knowledge affected the level of agreement about human involvement in GM similar to how it affected level of agreement about the safety of GM food. However, objective knowledge did not. Increases in *Perceived knowledge* decreased the probability of being in the Strongly Disagree, Disagree, and Neither Agree/Disagree categories and increased the probability of being in the Agree and Strongly Agree categories. Conversely, increases in *Actual knowledge* increased the probability of being in the Strongly Disagree, Disagree, and Neither Agree/Disagree categories and decreased the probability of being in the Agree and Strongly Agree categories. Thus, both subjective and objective knowledge affected the level of agreement that human involvement causes GW, however, subjective and objective knowledge also affected the level of agreement in the opposite direction. The signs of marginal effects for *Strong Democrat* were the same for all level of agreement categories for both GM and GW. *Strong Democrat* was less likely to be in the Strongly Disagree, Disagree, and Neither Agree/Disagree categories and more likely to be in the Agree and Strongly Agree categories. *Lean Democrat* was less likely to be in the Strongly Disagree category and more likely to be in the Agree. *Strong Republican* was somewhat agreeable about the safety of GM foods, but not about anthropogenic GW. *Strong Republican* was more likely to be in the Strongly Disagree, Disagree, and Neither Agree/Disagree categories and less likely to be in the Agree and Strongly Agree categories. Cognitive function, at least as defined by System 1 and System 2 and measured by the Cognitive Reflection Test, affected the probability of being in a level of agreement category for GW. Increases in *CRT* increased the probability of being in the Strongly Disagree, Disagree, and Neither Agree/Disagree categories and decreased the probability of being in the Agree and Strongly Agree categories. The only other independent variable that had significant marginal effects in the GW ordered probit model was *Bachelors*. A respondent who had obtained a Bachelor’s degree was less likely to be in the Strongly Disagree, Disagree, and Neither Agree/Disagree categories and more likely to be in the Agree and Strongly Agree categories.

**Table 4:** Marginal Effects from the Global Warming Ordered Probit Model.

	Dependent Variable: Level of Agreement				
Explanatory Variables	Strongly Disagree	Disagree	Neither Agree/Disagree	Agree	Strongly Agree
*Perceived knowledge*	-0.045***	-0.109***	-0.169***	0.150***	0.173***
*Actual knowledge*	0.010***	0.023***	0.036***	-0.032***	-0.037***
*Strong Democrat*	-0.016***	-0.046***	-0.088**	0.043***	0.107**
*Democrat*	-0.005	-0.013	-0.021	0.017	0.022
*Lean Democrat*	-0.010*	-0.027	-0.047	0.032**	0.053
*Independent*	0.004	0.010	0.015	-0.014	-0.015
*Lean Republican*	0.013	0.028	0.038	-0.042	-0.037
*Republican*	0.014	0.031	0.043	-0.046	-0.041*
*Strong Republican*	0.094***	0.135***	0.105***	-0.227***	-0.107***
*CRT*	0.007**	0.016**	0.025**	-0.022**	-0.026**
*Age*	0.000	0.000	0.000	-0.001	-0.001
*Bachelors*	-0.008**	-0.021**	-0.034*	0.027**	0.035*
*Female*	-0.005	-0.012	-0.018	0.016	0.018
*Income*	-0.001	-0.002	-0.004	0.003	0.004

Note: Estimates are from an ordered probit model using 955 observations and a log likelihood function of -1,107. Single, double, and triple asterisks (*, **, ***) indicate statistical significance at the 10%, 5%, and 1% level.

The null hypothesis that there was no significant relationship between levels of agreement and illusory correlations was rejected. The Pearson correlation coefficients between illusory correlation and level of agreement were -0.52 (*P* < 0.01) and -0.58 (*P* < 0.01) for GM and GW, respectively. Although it is unknown whether illusory correlations cause the formation of false hypotheses, or illusory correlations are formed to protect false hypotheses, there is a significant relationship between illusory correlations and false hypotheses.

Marginal effects from the multinomial logit model are displayed in Table 5. Increases in either *Perceived knowledge GM* or *Actual knowledge GM* increased the probability of being in both the *Do not disagree GM & GW* and *Do not disagree GM & disagree GW* categories and decreased the probability of being in the *Disagree GM & do not disagree GW* category. Increases in *Perceived knowledge GW* increased the probability of being in the *Do not disagree GM & GW* category and decreased the probability of being in the *Do not disagree GM & disagree GW* and *Disagree GM & GW* categories. While increases in *Actual knowledge GW* decreased the probability of being in the *Do not disagree GM & GW* category and increased the probability of being in the *Do not disagree GM & disagree GW* category. Similar to results found in the ordered probit models, subjective and objective knowledge affect beliefs in a similar manner for GM foods and in a different for GW. Also similar to the ordered probit models, Democrats were less likely to disagree with scientific consensus. *Combined Democrat* was more likely to be in the *Do not disagree GM & GW* category and less likely to be in the *Disagree GM & GW* category. *Independent* and *Combined Republican* were more likely to be in the *Do not disagree GM & disagree GW* category. Increases in *CRT* decreased the probability of being in the *Do not disagree GM & GW* category and increased the probability of being in both the *Disagree GM & do not disagree GW* and *Disagree GM & GW* categories. Thus, respondents who tend to be more analytical were more likely to disagree with scientists. Unsurprising, respondents with a college education were more likely to agree with scientists. A respondent who had obtained a Bachelor’s degree was more likely to be in the *Do not disagree GM & GW* category and less likely to be in the *Do not disagree GM & disagree GW* category. Lastly, female respondents were less likely to be in the *Do not disagree GM & GW* and *Do not disagree GM & disagree GW* categories and more likely to be in the *Disagree GM & do not disagree GW* category.

**Table 5:** Marginal Effects from the Multinomial Logit Model.

	Dependent Variables			
Explanatory Variables	*Do not disagree GM & GW*	*Disagree GM & do not disagree GW*	*Do not disagree GM & disagree GW*	*Disagree GM & GW*
*Perceived knowledge GM*	0.051**	-0.079***	0.036***	-0.007
*Perceived knowledge GW*	0.102***	-0.008	-0.051***	-0.043***
*Actual knowledge GM*	0.082***	-0.116***	0.035***	-0.001
*Actual knowledge GW*	-0.037*	-0.002	0.027***	0.012
*Combined Democrat*	0.094**	-0.059	0.025	-0.060***
*Independent*	-0.015	-0.030	0.052**	-0.006
*Combined Republican*	-0.059	-0.062	0.114***	0.007
*CRT*	-0.088***	0.056**	0.015	0.016*
*Age*	0.001	-0.002	0.000	0.000
*Bachelors*	0.081*	-0.032	-0.045**	-0.004
*Female*	-0.129***	0.163***	-0.032**	-0.002
*Income*	-0.003	0.004	-0.000	-0.001

Note: Estimates are from univariate probit models using 955 observations. Standard errors are reported in parenthesis. Single, double, and triple asterisks (*, **, ***) indicate statistical significance at the 10%, 5%, and 1% level.

## Conclusions

There is great uncertainty due to the challenges of escalating population growth and global warming. Along with the disagreement regarding policy implications, the issues are further complicated by the gap between science and public opinion. The ability of public science to contribute to these pressing issues will partly depend on public opinion. This study sought to provide a better understanding of factors associated with public opinion that opposes scientific consensus regarding the safety of GM foods and human involvement in GW.

The effects of knowledge on public opinion are complex and non-uniform across types of knowledge or issues. Perceived knowledge consistently affected opinions for both GM foods and GW. Individuals with greater perceived knowledge were more likely to agree about the safety of GM food and human involvement causing GW. Actual knowledge did not consistently affect opinions for either issue. Individuals with greater actual knowledge about GM foods were more agreeable about GM food safety; however, individuals with greater actual knowledge about GW were less agreeable about anthropogenic GW. Although, the latter finding appears to confirm the finding of previous research that concluded that greater actual knowledge is associated with being less agreeable about contentious issues [8], this finding was inconsistent for both issues. It is unclear why respondents with relatively higher actual knowledge about GW were more likely to disagree that human involvement causes GW. It could be that people who do not agree with science about GW may have sought information to be more knowledgeable about the subject. Public disagreement with science may be characterized as a deficiency in knowledge or understanding [7] and it has been suggested that increased communication can help to resolve these deficiencies [13,24]. However, it is not clear that providing the public with more information would have a desired outcome if the goal of increased communication is to decrease the gap between the public and science. Moreover, research that has examined the effects of information concluded that simply providing individuals with information is insufficient for changing behavior [31,32].

Whether human involvement causes GW continues to be a politically polarizing issue for individuals at the extreme ends of the political affiliation spectrum (Democrat vs Republican). Democrats were more likely to agree that human involvement is causing GW, and strong Republicans were most likely to form the exact opposite opinion. An interesting result was that respondents identifying as a strong Democrat or Democrat agreed that GM food was safe to consume, while Republicans did not. These results do not diminish claims that Republicans are science deniers. Giving more credence to claims that Democrats are more accepting of science was the result that Democrats was more likely to be in the joint categories of *Do not disagree GM & GW* and *Disagree GM & GW*. Referring back to the Anti-Reflexivity Thesis [20], it was expected that Republicans would be relatively more agreeable towards GM foods and Democrats should be relatively more agreeable to anthropogenic GW. While this was not the confirmed when examining agreement with the issues independently, there was confirmation of the Anti-Reflexivity Thesis when examining agreement with the issues jointly. Republicans were more likely to be in the joint *Do not disagree GM & disagree GW* category which is exactly what the Anti-Reflexivity Thesis postulated. Therefore, it is important to examine opinions for issues jointly as well as separately.

A relatively higher cognitive function, as measured by the Cognitive Reflection Test [27], was associated with beliefs that contradict the scientific community. This result indicated that people who rely more on analytical intelligence (System 2) were more likely to disagree with scientific consensus about GM food safety and human involvement in GW. Previous research has found that people who have a tendency to rely more on intuition (System 1) produce automatic judgments that are supported by intuitive belief-formation processes [33]. Thus, people who adopt a similar opinion as the scientific community may do so because it provides accessible explanations to questions that require a high level of expertise, whereas people who do not adopt a similar opinion may do so because of analytical processes. This conclusion would confirm Toplak, West, and Stanovinch [28] which concluded that CRT is a potent measure of miserly processing and tendency to heuristically triggered responses.

While previous research concluded that demographic characteristics were predictors of concerns about GM food and GW [11,14,16], the effects found within were scarce. Females were more likely to not be agreeable about the safety of GM foods, but there was no sex effect for GW. The finding for GW does not explicitly contradict previous research because the questions asked were different [11,16]. However, females were more likely to be in the joint *Disagree GM &Do not disagree GW* category which confirms the theory that women express greater concern about the safety of technological risk and the environment [34]. Individuals with a Bachelor’s degree were more likely not to be agreeable about anthropogenic GW, but there was no education effect for GM food. McCright [24] found that education was not significantly related to concern about GW being a serious threat in a respondent’s lifetime; however, education was significantly related to the agreement that human actions cause GW.

There appears to be a strong association between illusory correlations and disagreement with the scientific community. This study cannot conclude that decreasing illusory correlation would decrease disagreement. Nevertheless, efforts to decrease illusory correlations may be a more effective form of scientific communication than simply providing information, which has been found to be ineffective [31,32]. Future research may provide more insights into the causal relationship between illusory correlation and beliefs.

There are limitations to this analysis. Focus on agreement with science was placed on political affiliation, and while political affiliation is a signal of an individual’s worldview, it is not a perfect signal. Risk perceptions matter [35], and a consideration of attitudes towards risk may provide some insight about the gap between public opinion and science [36,37]. Furthermore, this analysis did not measure trust in corporations or government, which may affect agreement about both the safety of GM food and human involvement in GW.

## Supporting Information

S1 File Description of Survey Questions. (PDF)

S2 File Dataset used in Analysis. (CSV)
