# Supplementary material for: Examining the Gap between Science and Public Opinion about Genetically Modified Food and Global Warming
Source: PLoS One. 2016 Nov 9;11(11):e0166140. doi: 10.1371/journal.pone.0166140 (PMC5102371; doi:10.1371/journal.pone.0166140)
Supplement: S1 File — (PDF) [file pone.0166140.s001.pdf]

S1 File. Description of Survey Questions.

Questions used to create the dependent variables:

GM Question:

Q2 To what extent do you agree or disagree with the following statement? "Genetically modified crops are safe to eat."

- ❍ Strongly Disagree (1)
- ❍ Disagree (2)
- ❍ Neither Agree nor Disagree (3)
- ❍ Agree (4)
- ❍ Strongly Agree (5)

GW Question:

Q17 To what extent do you agree or disagree with the following statement? "The Earth is getting warmer because of human actions."

- ❍ Strongly Disagree (1)
- ❍ Disagree (2)
- ❍ Neither Agree nor Disagree (3)
- ❍ Agree (4)
- ❍ Strongly Agree (5)

Questions used for the independent variables:

Political Affiliation Question:

Q34 What is your political party affiliation?

- ❍ Strong Democrat (1)
- ❍ Democrat (2)
- ❍ Independent Lean Democrat (3)
- ❍ Independent (4)
- ❍ Independent Lean Republican (5)
- ❍ Republican (6)

- Strong Republican (7)
- I don’t know (8)
- Other (9)

Perceived Knowledge GM Question:

Q4 To what extent do you agree or disagree with the following statement? "Scientific research supports my views about the safety of genetically modified crops."

- Strongly Disagree (1)
- Disagree (2)
- Neither Agree nor Disagree (3)
- Agree (4)
- Strongly Agree (5)

Perceived Knowledge GW Question:

Q19 To what extent do you agree or disagree with the following statement? "Scientific research supports my views about human activity and global warming."

- Strongly Disagree (1)
- Disagree (2)
- Neither Agree nor Disagree (3)
- Agree (4)
- Strongly Agree (5)

Actual Knowledge GM Questions:

Q8 Is the following statement true or false? "Ordinary tomatoes do not contain genes while genetically modified tomatoes do."

- True (1)
- False (2)

Q9 Is the following statement true or false? "By eating a genetically modified fruit a person's genes could become modified."

- True (1)

❍ False (2)

Q10 Is the following statement true or false? "Genetically modified animals are always bigger than ordinary ones."

❍ True (1)

❍ False (2)

Actual Knowledge GW Questions:

Q23 Is the following statement true or false? "Climate often changes from year to year."

❍ True (1)

❍ False (2)

Q24 Is the following statement true or false? "Changes in local weather indicate changes in climate."

❍ True (1)

❍ False (2)

Q25 Is the following statement true or false? "The greenhouse effect is the same thing as global warming."

❍ True (1)

❍ False (2)

CRT Questions:

Q41 A bat and a ball cost $1.10 in total. The bat costs $1.00 more than the ball. How much does the ball cost?

Q42 If it takes 5 machines 5 minutes to make 5 widgets, how long would it take 100 machines to make 100 widgets?

Q43 In a lake, there is a patch of lily pads. Every day, the patch doubles in size. If it takes 48 days for the patch to cover the entire lake, how long would it take for the patch to cover half the lake?

Illusory Correlation GM Questions:

Q5 To what extent do you agree or disagree with the following statement? "Genetically modified crops have caused an increase in food allergies."

- Strongly Disagree (1)
- Disagree (2)
- Neither Agree nor Disagree (3)
- Agree (4)
- Strongly Agree (5)

Q6 To what extent do you agree or disagree with the following statement? “Genetically modified crops have caused an increase in incidence of Autism.”

- Strongly Disagree (1)
- Disagree (2)
- Neither Agree nor Disagree (3)
- Agree (4)
- Strongly Agree (5)

Q7 To what extent do you agree or disagree with the following statement? “Genetically modified crops were invented by Monsanto and are ruining humanity.”

- Strongly Disagree (1)
- Disagree (2)
- Neither Agree nor Disagree (3)
- Agree (4)
- Strongly Agree (5)

Illusory Correlation GW Questions:

Q20 To what extent do you agree or disagree with the following statement? “The Earth is not warming, the Earth is actually cooling.”

- Strongly Disagree (1)
- Disagree (2)
- Neither Agree nor Disagree (3)
- Agree (4)
- Strongly Agree (5)

Q21 To what extent do you agree or disagree with the following statement? “The warming of the Earth is just a natural cycle.”

- Strongly Disagree (1)
- Disagree (2)
- Neither Agree nor Disagree (3)
- Agree (4)
- Strongly Agree (5)

Q22 To what extent do you agree or disagree with the following statement? “Global warming is a conspiracy to redistribute wealth from the United States to other countries.”

- Strongly Disagree (1)
- Disagree (2)
- Neither Agree nor Disagree (3)
- Agree (4)
- Strongly Agree (5)

Demographic Questions:

Q45 What is your current age in years?

Q47 Have you obtained a Bachelor’s degree from a university or college??

- Yes (1)
- No (2)

Q44 What is your gender?

- Male (1)
- Female (2)

Q34 What is your approximate annual household income before taxes in 2010?

- Less than $20,000 (1)
- $20,000 to $39,999(2)
- $40,000 to $59,999 (3)
- $60,000 to $79,999 (4)
- $80,000 to $99,999 (5)
- $100,000 to $119,999 (6)

- $120,000 to $139,999 (7)
- $140,000 or more (8)
